# Biosorption of Cadmium and Manganese Using Free Cells of *Klebsiella* sp. Isolated from Waste Water

**DOI:** 10.1371/journal.pone.0140962

**Published:** 2015-10-27

**Authors:** Yunnan Hou, Keke Cheng, Zehua Li, Xiaohui Ma, Yahong Wei, Lei Zhang, Yao Wang

**Affiliations:** 1 College of Life Sciences, Northwest A&F University, Yangling, Shaanxi, China; 2 State Key Laboratory of Crop Stress Biology for Arid Areas, Northwest A&F University, Yangling, Shaanxi, China; 3 Biomass Energy Center for Arid and Semi-Arid Lands, Northwest A&F University, Yangling, Shaanxi, China; Glasgow University, UNITED KINGDOM

## Abstract

In the present study, we evaluated a bacterium that was isolated from waste water for its ability to take up cadmium and manganese. The strain, identified both biochemically and by its 16S rRNA gene sequence as *Klebsiella*, was named Yangling I2 and was found to be highly resistant to heavy metals. Surface characterization of the bacterium via SEM revealed gross morphological changes, with cells appearing as biconcave discs after metal exposure rather than their typical rod shape. The effects of pH, temperature, heavy metal concentration, agitation and biomass concentration on the uptake of Cd(II) and Mn(II) was measured using atomic absorption spectrophotometry. The results showed that the biosorption was most affected by pH and incubation temperature, being maximized at pH 5.0 and 30°C, with absorption capacities of 170.4 and 114.1 mg/g for Cd(II) and Mn(II), respectively. Two models were investigated to compare the cells’ capacity for the biosorption of Cd and Mn, and the Langmuir model based on fuzzy linear regression was found to be close to the observed absorption curves and yield binding constants of 0.98 and 0.86 for Cd and Mn, respectively. This strain of *Klebsiella* has approximately ten times the absorption capacity reported for other strains and is promising for the removal of heavy metals from waste water.

## Introduction

Heavy metal pollution is a global concern in the environmental field. Heavy metals, such as cadmium, copper and manganese, are among the most common pollutants found in industrial effluents (such as from mining, the surface finishing industry, energy and fuel production, and electric appliance manufacturing) [1]. Even at low concentrations, these metals are toxic to organisms, including humans.

Cadmium is a metal with a small biological demand that is only slightly degraded in the environment. Additionally, it has many common industrial uses, including battery production [2], coatings, and electroplating [3].Cadmium has been well recognized for its negative effect on the environment, where it accumulates readily in living systems [4]. Adverse health effects due to cadmium are well documented, and this metal has been reported to cause renal disturbances, lung insufficiency, bone lesions, cancer and hypertension in humans [5,6]. Although manganese is an essential trace nutrient in all known forms of life [7], higher levels of exposure to manganese in drinking water are associated with increased intellectual impairment and reduced intelligence quotients in school-age children [8,9].

Methods for removing heavy metals from solution consist primarily of physical, chemical and biological technologies. Conventional methods for removing metal ions from aqueous solution include chemical precipitation, filtration, ion exchange, electrochemical treatment, membrane technologies, absorption on activated carbon, and evaporation [10]. However, chemical precipitation and electrochemical treatment are ineffective, especially when the metal ion concentration in the aqueous solution is between 1 and 100 mg L^-1^. These techniques also produce large quantities of sludge that are very difficult to treat [11]. Ion exchange, membrane technologies and activated carbon absorption are extremely expensive when treating a large amount of water or when treating waste water containing heavy metals at low concentrations [12].

Biosorption can be defined as the removal of metal or metalloid species, compounds and particulates from solution by biological material [13]. Biosorption of heavy metals is one of the most promising technologies for the removal of heavy metals from waste water and is a potential alternative to conventional processes for metal removal. If the biomass employed is a waste material, then biosorption represents a cheap alternative to conventional processes because of a low-cost sorbent material can be used [14].

Interest in the development of metal removal by biosorption using microorganisms is evident in the recent literature, and the ability of microbial biomass to accumulate and remove heavy metals from water has been widely reported [15]. Manasi et al. [16] recently reported that *Halomonas BVR 1* isolated from an electronic industry effluent was able to absorb cadmium. Another report used *Pseudomonas aeruginosa* cells to bioabsorb manganese [17]. Studies involving the use of *Aspergillus niger* and *Saccharomyces cerevisiae* [18] have reported the high efficiency of manganese biosorption by these species. After equilibration times of 60 and 20 min, respectively, *Aspergillus niger* and *Saccharomyces cerevisiae* were shown to exhibit manganese biosorption rates of 19.34 and 18.95 mg/g. Not only bacteria can be used in biosorption; fungi, yeasts and eukaryotes have also been shown to be able to absorb heavy metals from solution [19]. Luna et al. [20] investigated the competitive biosorption of cadmium and zinc ions by *Sargassum filipendula* biomass single and binary systems. Non-living cells of the fungus *R*. *arrhizus* and the green alga *Schizomeris leiblenii* were studied by Ozer et al. [21] for their capacity to absorb up to 100 mg/g Cd(II).

Compared with the currently available studies of biosorption, *Klebsiella* sp. Yangling I2 has the unique abilities to resist and absorb heavy metals, thus opening the door to the removal of toxic ions from waste water using this organism.

Due to the serious pollution and the damage to mankind caused by cadmium and to this organism’s unique resistance to manganese, the aim of this study was to show the ability of cells of *Klebsiella* sp. Yangling I2 to bioadsorb cadmium and manganese.

## Materials and Methods

### Isolation and identification of bacteria from waste water

The waste water samples (10 mL) were collected from Common Effluent Treatment (CET) plant located in Xi’an, China. No specific permissions were required to access the above public area. We confirmed that the field studies did not involve endangered or protected species. The collected samples contained various metal ions and were stored at 4°C before analysis. Analytical-grade chemicals were used for the growth of the microorganisms, and bacterial strains were isolated from the effluents using Murashige and Skoog medium (K_2_HPO_4_ 1.6 g, KH_2_PO_4_ 0.4 g, NaNO_3_ 0.4 g, CaCl_2_ 0.02 g, MgSO_4_ 0.2 g, FeSO_4_·7H_2_O 0.01 g, and 50 ppm ibuprofen). To isolate strains, the serially diluted effluents were plated onto the same agar plates described above. The inoculated plates were incubated at 30°C for 48 h. After the incubation, the distinct colonies were isolated from each plate.

The strain was analyzed through morphological (Gram reaction, motility) and biochemical characterization, including carbohydrate fermentation, gelatin liquefaction test, starch hydrolysis test, and oxidation fermentation test in accordance with Bergey’s Manual of Determinative Bacteriology, as well molecular identification according to the 16S rRNA sequence. Amplification of 16S rRNA was carried out using universal primers (27 F 5’-AGAGTTTGATCMTGGCTCG-3’ and 1492 R 5’-GGTTACCTTGTTACGACTT-3’) with genomic DNA as a template [22,23]. The purified PCR products were sequenced by Sangon Biotech (Shanghai, China). BLAST was used to find similar sequences in the GenBank database (Nucleotide Blast), followed by multiple sequence alignment and phylogenetic tree construction in MEGA 5.0 using the neighbor-joining method.

### Growth of *Klebsiella* sp. Yangling I2 strain with different heavy metals

#### Determination of the minimum inhibitory concentration (MIC) of heavy metals

The minimum inhibitory concentration (MIC) of heavy metals was performed as follows: single clone growth was achieved on LB (Luria-Bertani Culture) agar plates, and the lowest concentration that prevented the bacterial growth was defined as the MIC. The resistant isolate was incubated in 50 mL of LB liquid medium (5 g of yeast extract, 10 g of casein enzymic hydrolysate, and 10 g of NaCl) to log phase at 30°C with shaking. Then, it was inoculated onto LB agar plates with different concentrations of CdSO_4_, ZnSO_4_, CuSO_4_·5H_2_O, Pb(NO_3_)_2_, K_2_Cr_2_O_7_, FeCl_3_, and MnCl_2_·4H_2_O. The concentrations of heavy metals were initially 100 mM and were diluted from 1X to 100X. After 2 days of incubation at 30°C, the metal MIC values were estimated in terms of the first dilution at which no single clone grew.

#### Growth curve

The strain was incubated in 50 mL of LB liquid medium (5 g of yeast extract, 10 g of peptone, and 10 g of NaCl) containing 1, 2, 3, or 4 mM of Cd(II) and 5, 10, 15 or 20 mM of Mn(II) at 30°C with shaking at 120 rpm. The optical density was measured at 600 nm (Telecomp, Shanghai, China) every 2 hours.

### SEM Analysis

Ten milliliters of LB culture of the resistant isolate containing 4 mM Cd(II) and 20 mM Mn(II) was incubated at 5,000 rpm at 4°C for 10 min. The pellet cells were fixed for 24 h in 3% glutaraldehyde solutions, followed by dehydration with a graded series of ethanol (50, 60, 70, and 80% and absolute, 15 min each) and drying under a CO_2_ atmosphere for 20 min using a critical point dryer (K850, Emitech, East Grinstead, UK). The samples were mounted on a stainless steel slab and covered with a thin layer of platinum under vacuum. The scanning electron microscopy (SEM) images and the energy dispersive spectral (EDS) analyses of the cells were obtained using a SEM instrument (S-4800, Hitachi, Tokyo, Japan).

### Preparation of Biomass and Ion Solution

Test solutions (50 mL each) containing Cd(II) and Mn(II) were prepared from CdSO_4_ and MnCl_2_·4H_2_O in a graded series from 0.5 mM to 20 mM. The initial pH of each test solution was adjusted to the appropriate value by 1 M HCl or 1 M NaOH before the addition of biomass.

Using LB liquid medium, stationary stage cultures were harvested by centrifugation (8,000 rpm, 20 min). The cells were washed two times with PBS buffer and suspended in PBS buffer (1 M, pH 7.2). The concentrations of the biosorbents were calculated and expressed in terms of grams (dry weight) per liter after 18-mL biomass samples were oven-dried at 60°C.

### Cd(II) and Mn(II) biosorption

To determine the initial pH for absorption, test solutions containing 2 mM Cd(II) and 10 mM Mn(II) with pH values ranging from 3.5 to 5.5 and 4.0 to 6.0, respectively, were used. Organisms were added at a concentration of 1 g/L. The cell-metal solutions were agitated at 120 rpm and 30°C, for 20 h or 26 h. After incubation, the supernatants were collected by centrifugation at 8,000 rpm for 20 min. Using a suitable dilution, the amount of residual ion in the solutions was determined using an atomic absorption spectrometer (AAS) (Z-2000; Hitachi, Tokyo, Japan).

Different initial ion concentrations (0.5–4 for Cd(II) and 3–20 mM for Mn(II), biomass densities (1–5 g/L), agitation speed (60–180 rpm) and incubation temperature (20–40°C)) were investigated using Cd(II) and Mn(II) test solutions containing 1 g/L biomass, 2 mM Cd(II) and 10 mM Mn(II) at an initial pH based on the results reported above.

The ion supernatants were measured as described above. All experiments were performed in triplicate. The absorption capacity (Q_e_) and the removal ratio (R_e_%) were evaluated by the equilibrium described by Eqs (1) and (2): [24,25]

Qe=Ci−Cfv*M (1)

Re(%)=Ci−CfCi*100% (2)

where

Q_e_=Metal uptake (micrograms of metal per gram of biosorbent)

V = Liquid sample volume (milliliters)

C_i_=Initial concentration of the metal in the solution (milligrams per liter)

C_f_=Final concentration of the metal in the solution (milligrams per liter)

M=Amount of added biosorbent on a dry basis (milligrams)

### Determination of equilibrium time for Cd(II) and Mn(II) absorption

The effect of contact time on the ion uptake of *Klebsiella* sp. Yangling I2 was investigated using solutions of 2 mM CdSO_4_ and 10 mM MnCl_2_ at pH values of 5.5 and 5, respectively. Samples were taken at designated time points (5, 10, 15, 20, 25, 30, 40, 50, 60, 80, 100, 120, 150, 180, 240, and 300 min). The supernatants were collected by centrifugation at 8,000 rpm for 20 min and used to determine the amounts of the residual Cd and Mn ions by AAS.

### Biosorption isotherms

Biosorption isotherms characterized by certain constant values indicate the surface properties and affinity of the biosorbents [26] and can be used to compare the capacities for biosorption of various heavy metals. Metal uptake by organisms can be described in terms of two stages: an initial rapid stage (passive uptake) and a much slower stage (active uptake) [27]. The biosorption isotherms of Cd(II) and Mn(II) were investigated using two isotherm models: the Langmuir and Freundlich isotherm models.

#### Langmuir isotherm

The Langmuir isotherm is used to examine the absorption of gases on a solid surface, and sorption is considered to be a chemical phenomenon. This isotherm has been successfully applied to many pollutant biosorption processes and is the most widely used isotherm for the biosorption of a solute from a liquid solution [28].

#### Freundlich isotherm

The Freundlich isotherm is applied under the assumption of a heterogeneous absorption surface and active sites with different energies [28]. The model is represented in Eq (3): [29,30]

qe=KfCe1n (3)

where K_f_ is a Freundlich constant relating the binding capacity and 1/n is an empirical parameter relating the biosorption intensity, which varies with the heterogeneity of the biosorbents. An efficient absorption process yields a Freundlich constant n between 1 and 10. A high value of n implies a stronger interaction between the adsorbent cell surface and divalent metals.

## Result and Discussion

*Klebsiella* sp. Yangling I2 was named for the place where it was found, and the sequence generated in this study was submitted to the NCBI GenBank database under the accession number JX196956.1. The strain was analyzed in accordance with Bergey’s Manual of Determinative Bacteriology, and molecular identification was achieved according to the 16S rRNA sequence, which showed 97% homology.

### The tolerance of *Klebsiella* sp. Yangling I2 to different heavy metals

To examine the tolerance of *Klebsiella* sp. Yangling I2 to different metals, the cells were cultivated in nutrient broth with Cd(II), Zn(II) Mn(II), Fe(III), Cu(II) and Cr(VI). The organism was able to survive at metal concentrations as high as 80 mM for Mn(II), 6 mM for Zn(II) and Cd(II), 5 mM for Fe(III), 2 mM for Cu(II) and 0.1 mM for Cr(VI).

### Growth curve

The ability of *Klebsiella* sp. Yangling I2 to tolerate cadmium and manganese was higher than for other metals and strains. Therefore, these two metals were selected for further metal absorption studies.

**Fig 1:**
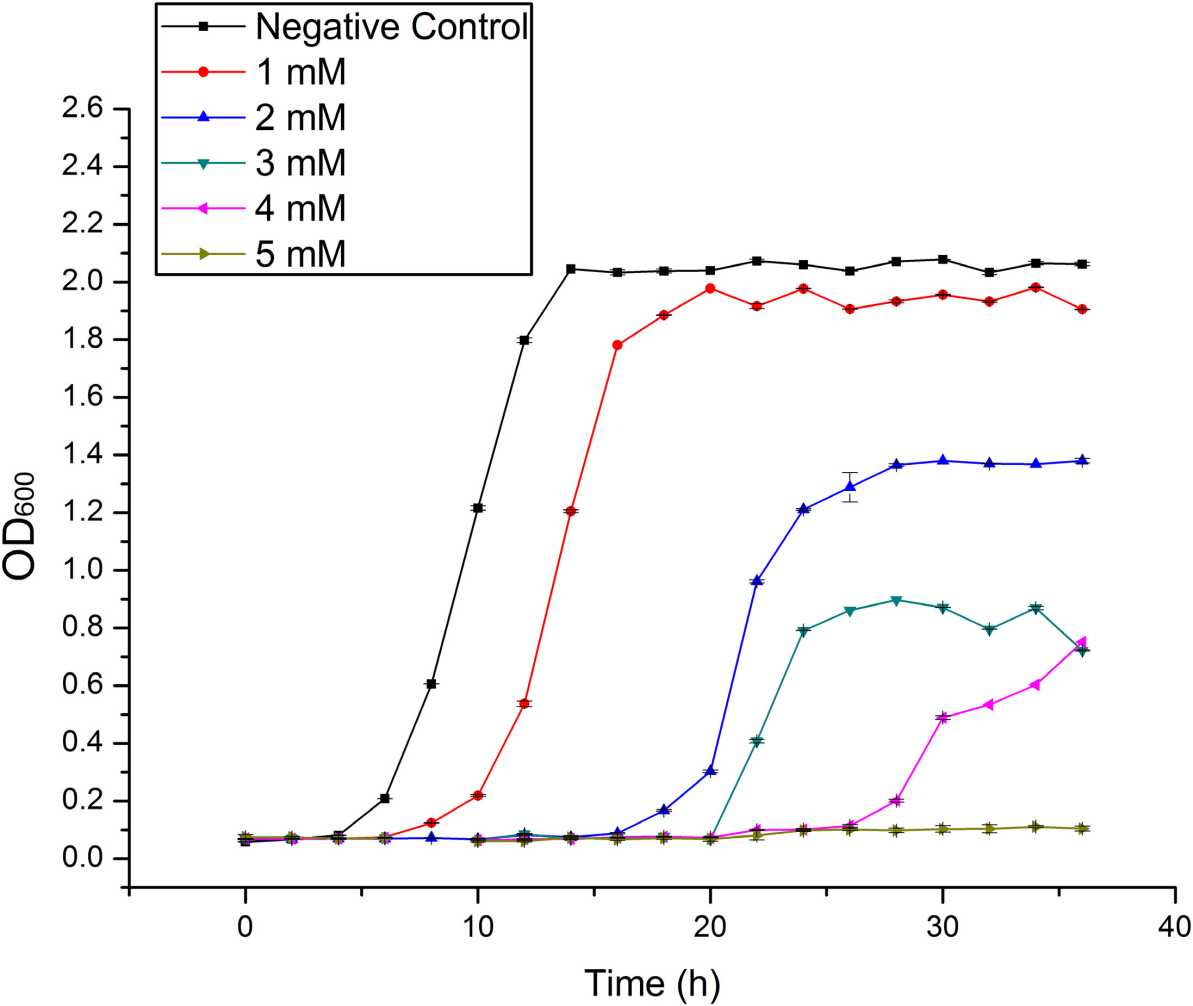
The growth curves of *Klebsiella* sp. Yangling I2 in the presence of different initial concentrations of Cd(II). Initial pH = 5.0, biomass concentration = 1.0 g/L, and contact time = 26 h.

**Fig 2:**
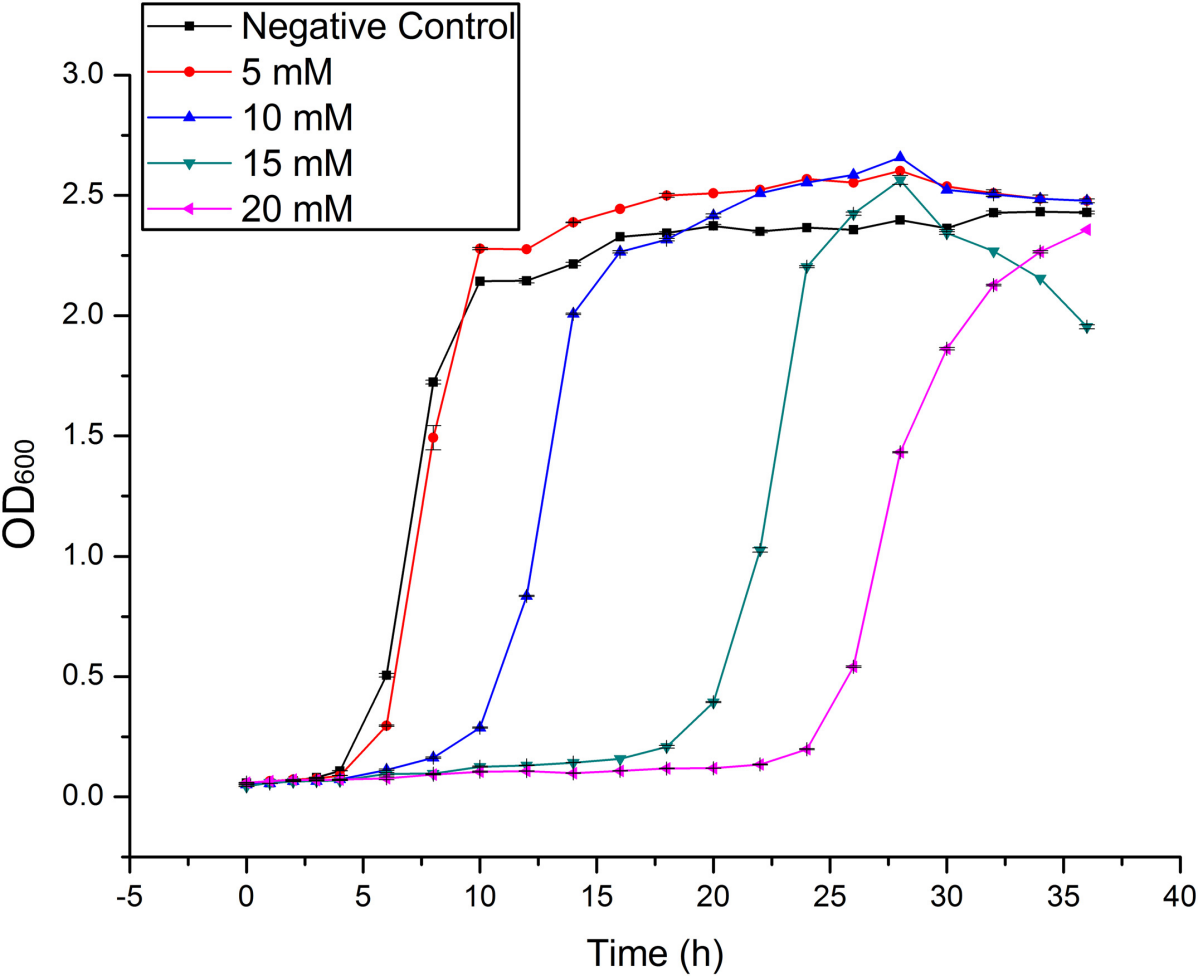
The growth curves of *Klebsiella* sp. Yangling I2 in the presence of different initial concentrations of Mn(II). Initial pH = 5.0, biomass concentration = 1.0 g/L, and contact time = 26 h.

Figs 1 and 2 show the growth curve of *Klebsiella* sp. Yangling I2 in the presence of heavy metals. The presence of heavy metal ions interferes with the growth of organisms and delays the log phase. There is evidence that the cell metabolism is inhibited mainly by ions transported into the cell, and perhaps also by ions adsorbed on the outer surface [31].

Because it resists high concentrations of cadmium and manganese, *Klebsiella* sp. Yangling I2 might be capable of removing these ions from effluent water heavily polluted by them.

### Morphology of the adsorbent according to SEM analysis

**Fig 3:**
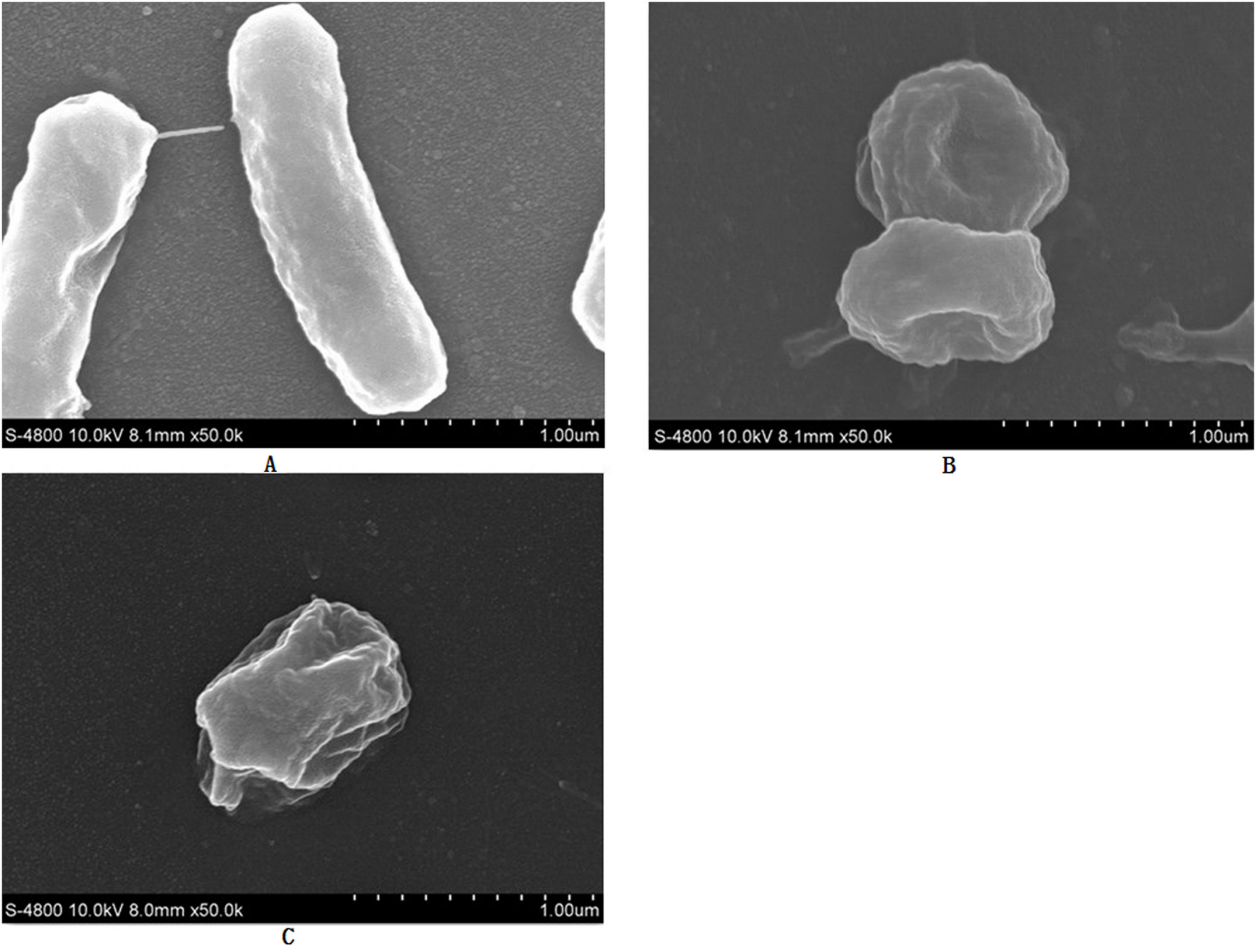
**(A)** SEM images of *Klebsiella* sp. Yangling I2, **(B)** *Klebsiella* sp. Yangling I2 equilibrated with 4mM Cd(II), and **(C)** *Klebsiella* sp. Yangling I2 equilibrated with 20mM Mn(II).

Electron microscopic examination of *Klebsiella* sp. Yangling I2 before and after metal removal was undertaken to locate the active sites of the cell wall. Scanning electron microscopy (SEM) at 50,000X showed that the *Klebsiella* sp. Yangling I2 was morphologically rod shaped, as demonstrated in Fig 3A, with lengths and diameters reaching 14 μm and 3 μm, respectively. The small size of *Klebsiella* sp. Yangling I2, with its estimated surface area of 146 μm^2^, gives it a large contact surface [32], which should facilitate interaction with metals, and thus, biosorption [28,33].

After equilibration with a metal solution, the cell wall, shape and size of the bacteria changed. The length and size of the cell decreased (Fig 3B and 3C), and the cells began to appear as biconcave discs after exposure to 4 mM Cd(II). These bacteria typically exhibit a rod shape (Fig 3B), and this changes suggests that the metal ions were entrapped in the extracellular polymeric substances of *Klebsiella* sp. Yangling I2, thus causing deformation of and damage to the cell surface during Cd(II) and Mn(II) absorption.

### Effect of Parameters on single Ion Biosorption

#### The effect of pH on biosorption

**Fig 4:**
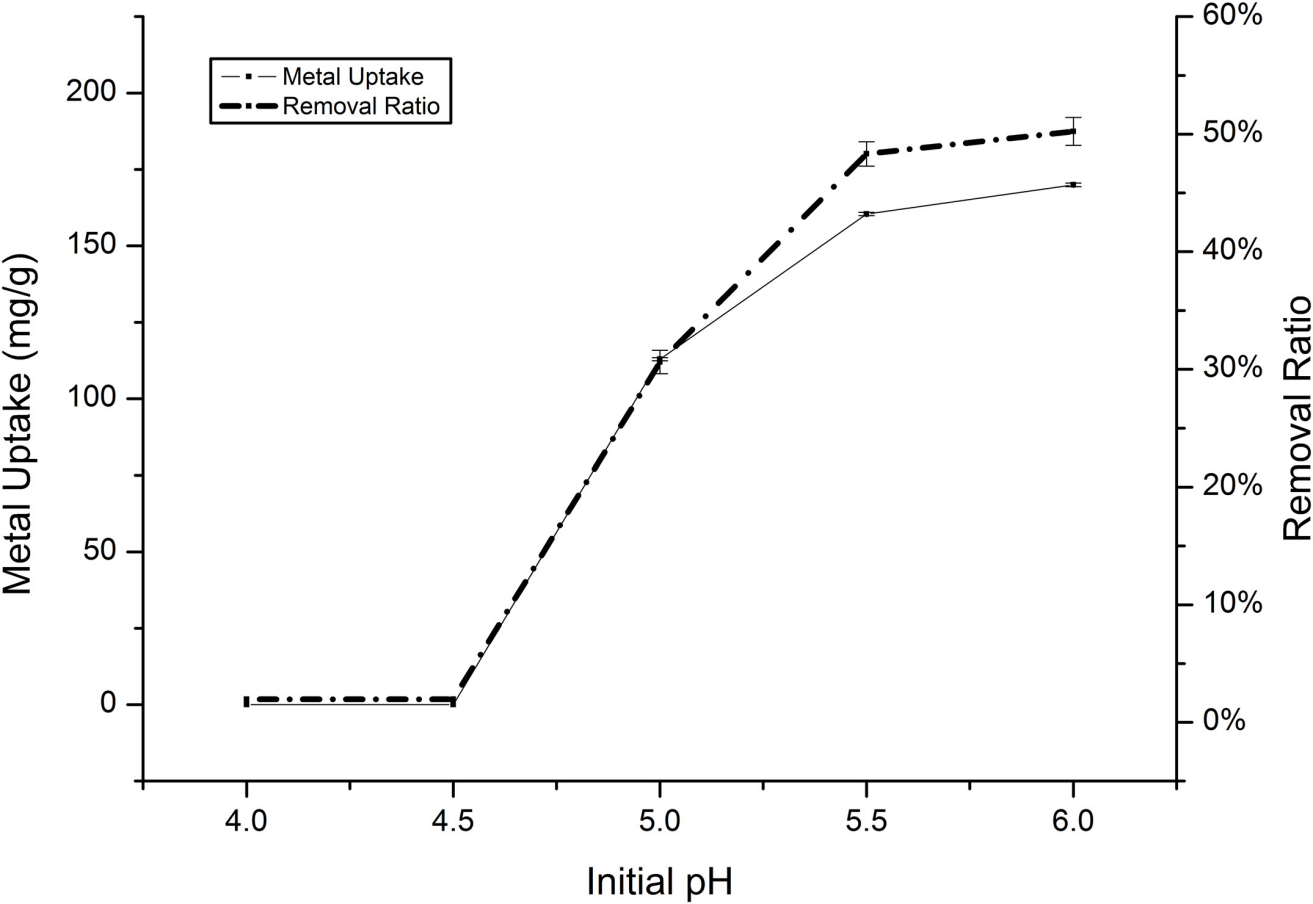
Effect of pH on the absorption of Cd(II) by *Klebsiella* sp. Yangling I2. Initial ion concentration = 2 mM, biomass concentration = 1.0 g/L, and contact time = 26 h.

**Fig 5:**
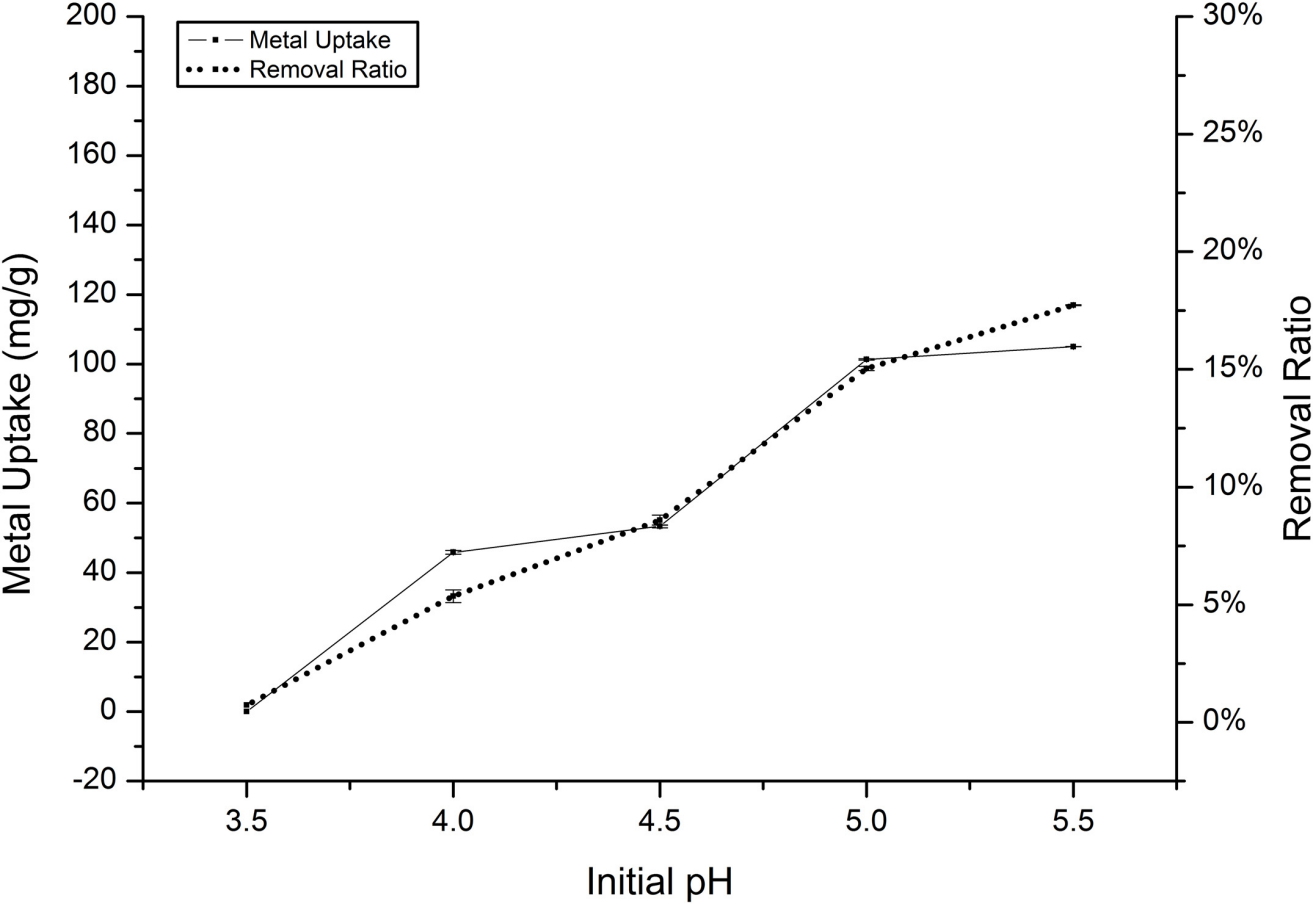
Effect of pH on the absorption of Mn(II) by *Klebsiella* sp. Yangling I2. Initial ion concentration = 10 mM, biomass concentration = 1.0 g/L, and contact time = 20 h.

Previous investigations into heavy metal biosorption have shown that the pH value is an important factor for ion absorption, although pH values higher than 6.0 were not tested to prevent the precipitation of insoluble cadmium and manganese hydroxide [34,35]. The significant effect of pH on the absorption of Cd(II) and Mn(II) by *Klebsiella* sp. Yangling I2 was obvious. The uptake capacities of the two metals generally exhibited a similar trend, with higher pH values leading to a higher metal uptake. Regarding to Cd(II), the biosorption capability increased gradually as the pH increased, although the absorption capability of this species is higher than that of Mn(II), with a maximum value of 169.94 mg/g (Fig 4). However, *Klebsiella* sp. Yangling I2 was very difficult to grow in solutions with pH values of less than 4.5, which might be because of the competition between hydrogen and metal ions for the sorption sites on the biomass surface [36,37]. For Mn(II), the biosorption capability increased gradually in the range of pH 3.5 to 5.5, eventually reaching a maximum value of 105.08 mg/g. The curve of the removal ratio increased, following the trend shown by biosorption (Fig 5).

The dependence of metal uptake on pH is related to both the surface functional groups on the cell walls of the biomass and the metal chemistry in solution. In a solution with low pH, the positively charged hydrogen ions may compete with metal ions to bind to the ligands on the cell wall. Increased pH results in increased availability of ligands for metal ion binding, thus enhancing biosorption [38]. In order to test this theory, 1M HCl was added into the culture medium which cells were already finished the process of biosorption. The concentration of metal ions was increased when more HCl was added into the solution.

#### The effect of incubation temperature on biosorption

**Fig 6:**
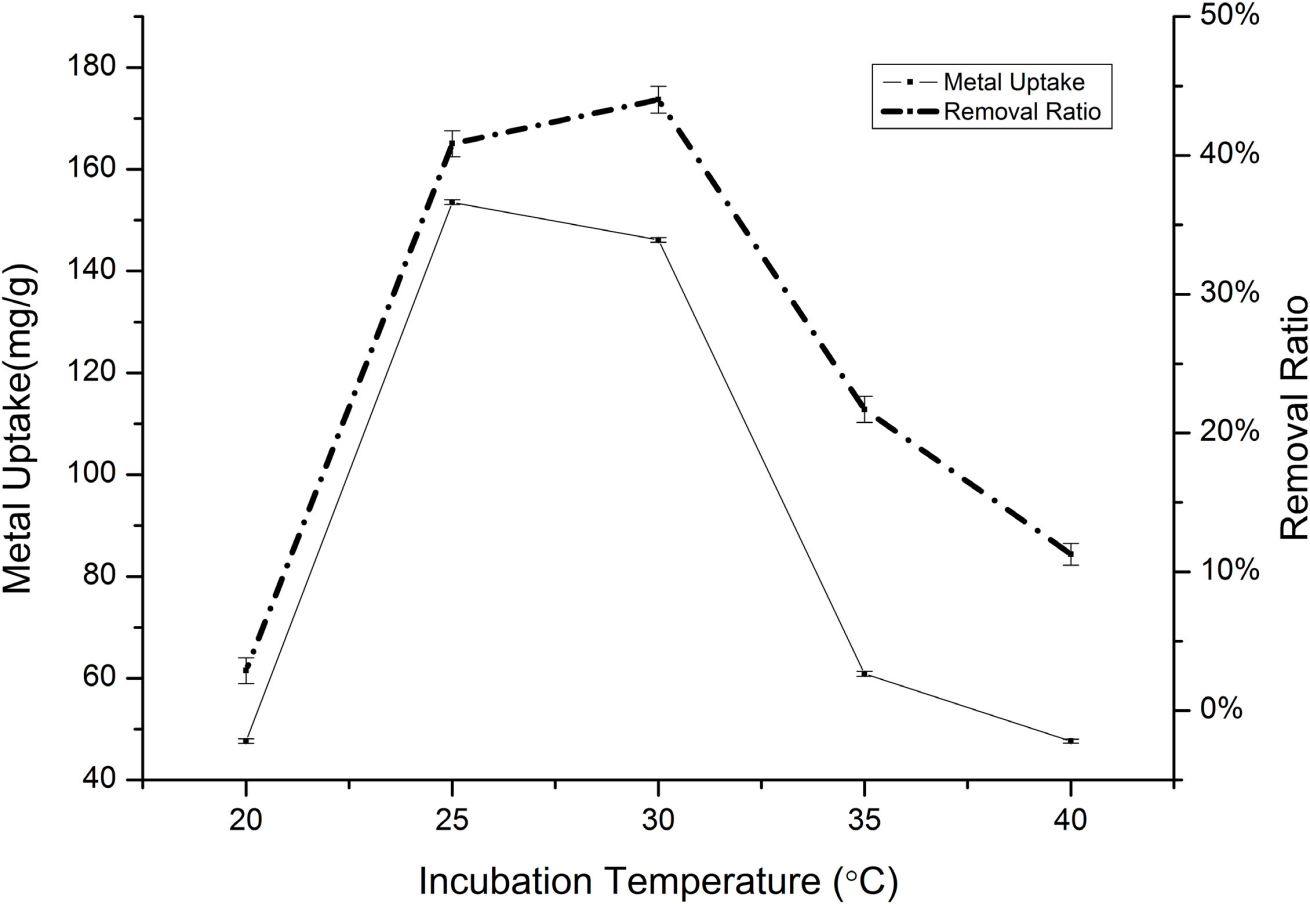
Effect of incubation temperature on the absorption of Cd(II) by *Klebsiella* sp. Yangling I2. Initial ion concentration = 10 mM, initial pH = 5.0, and contact time = 26 h.

**Fig 7:**
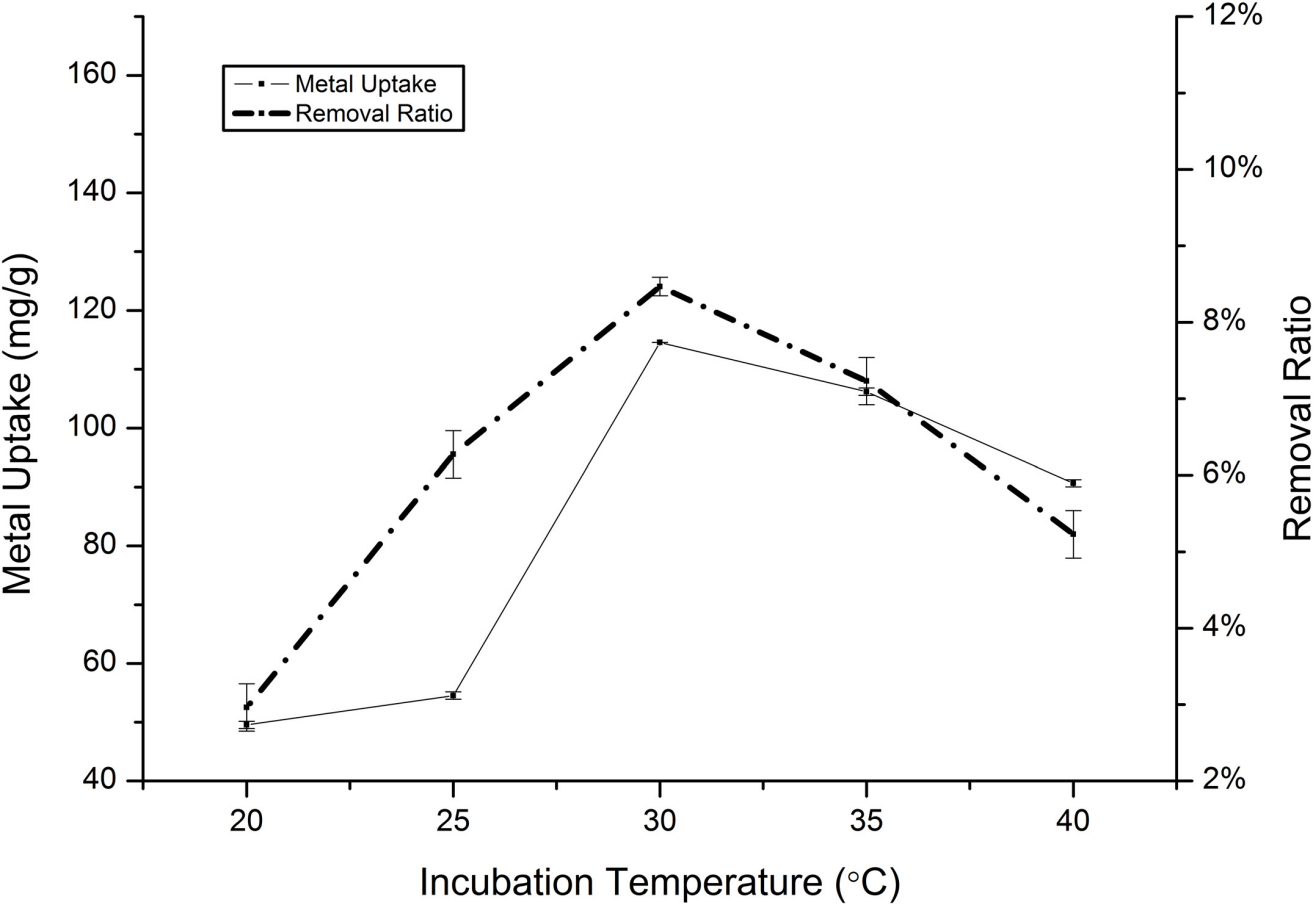
Effect of incubation temperature on the absorption of Mn(II) by *Klebsiella* sp. Yangling I2. Initial ion concentration = 10 mM, initial pH = 5.0, and contact time = 20 h.

To study the effect of the incubation temperature on the binding force between glycoproteins on the membrane and heavy metal ions, we selected the following temperatures: 20, 25, 30, 35 and 40°C. The results demonstrated that an increase in the temperature in the range 20–25°C resulted in an increase in the cadmium-sorption capacity at equilibrium: 47.67 mg/g at 20°C and 153.52 mg/g at 25°C (Fig 6). At temperatures exceeding 25°C, the sorption capacities of both cadmium and manganese decreased, reaching a maxium value of 114.56 mg/g during the temperature exceeded from 25 to 30°C for cadmium (Fig 7). Within the temperature range investigated, it is possible to conclude that an increase in temperature is followed by an increase in the diffusivity of the ion [39]. Therefore, like chemical reactions, there is an optimal temperature at which the ratio of sorption to desorption is maximal, as is supported by our results.

#### The effect of heavy metal concentration on biosorption

**Fig 8:**
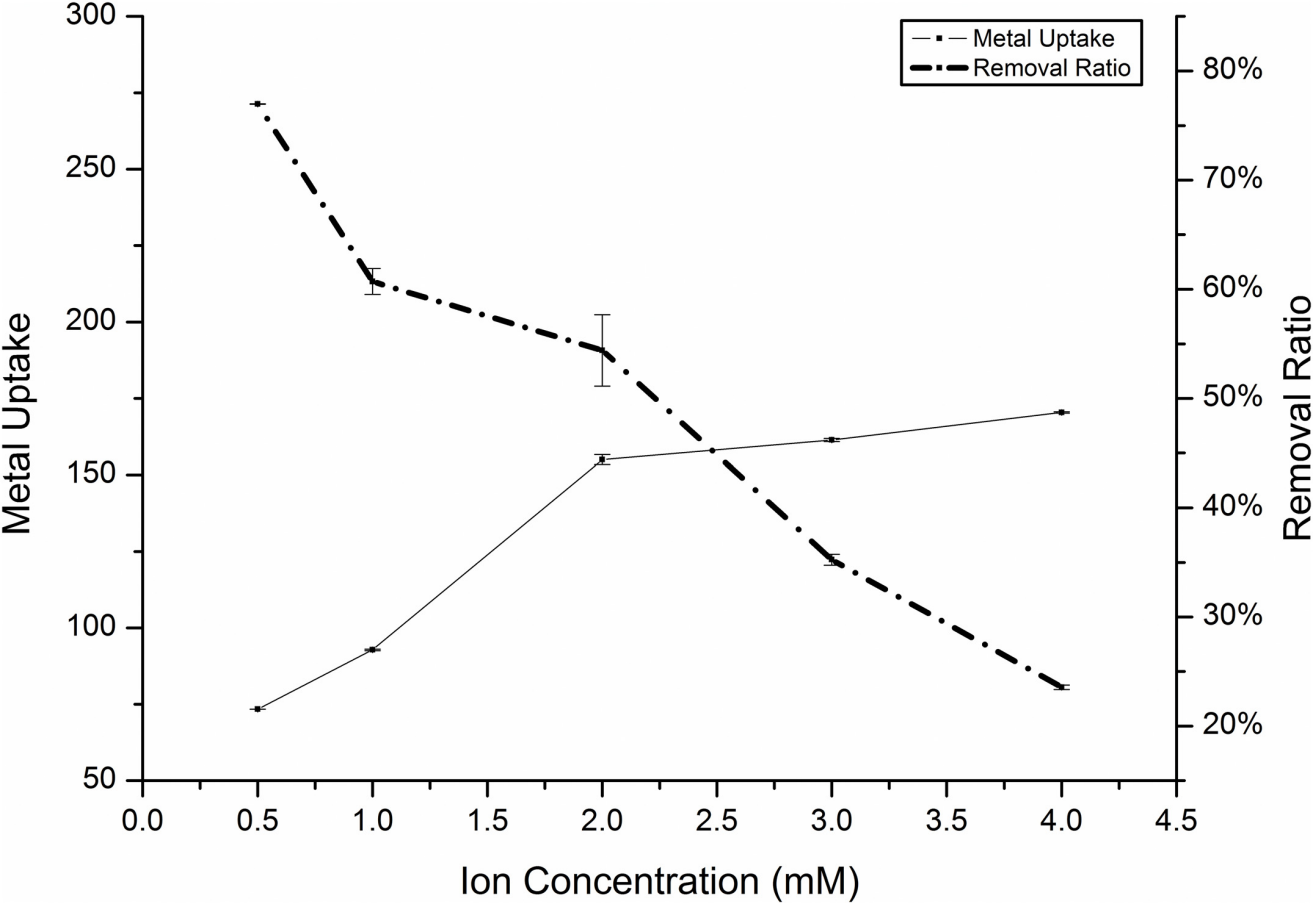
Effect of initial ion concentration on the absorption of Cd(II) by *Klebsiella* sp. Yangling I2. Initial pH = 5.5, biomass concentration = 1.0 g/L, and contact time = 26 h.

**Fig 9:**
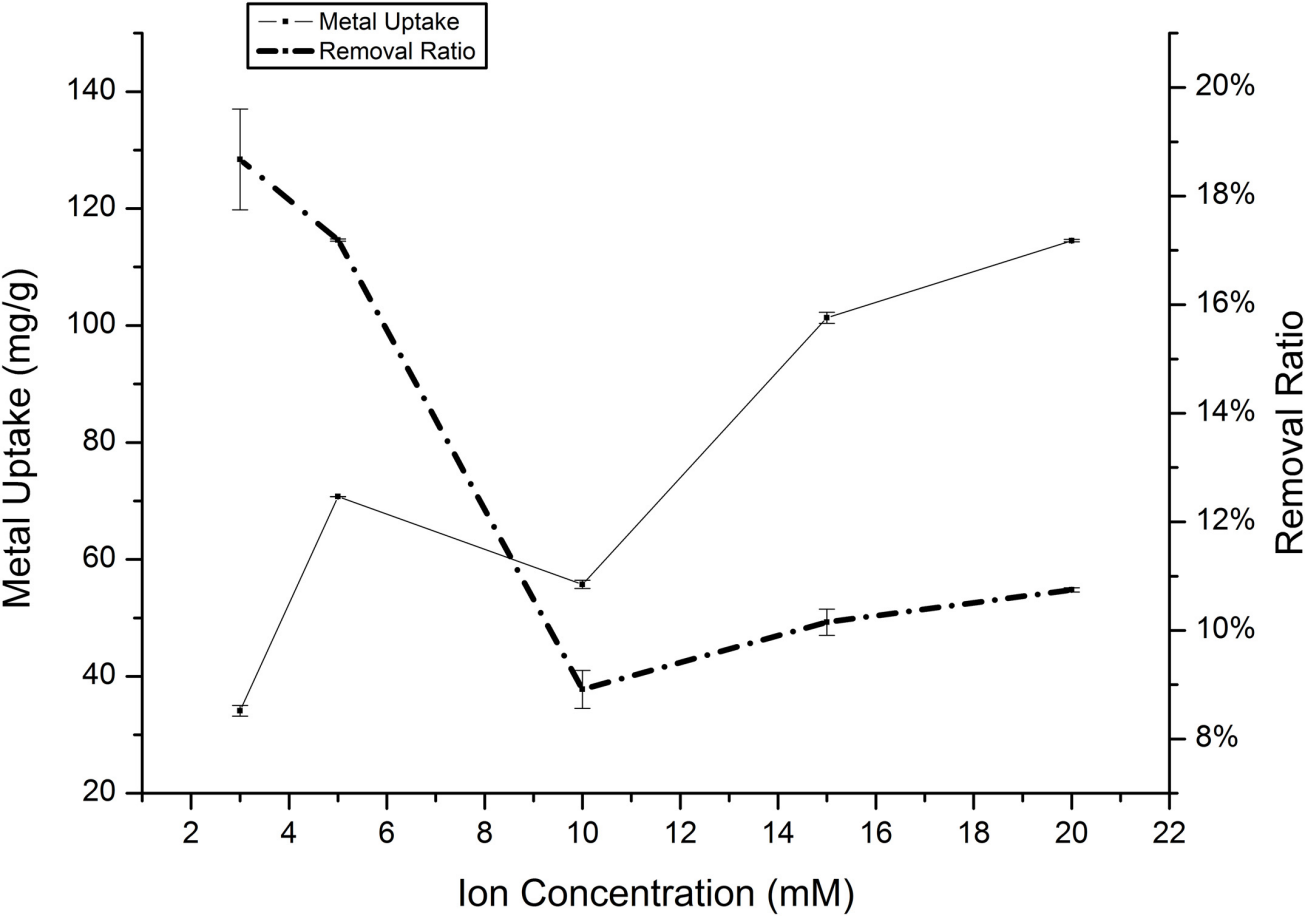
Effect of initial ion concentration on the absorption of Mn(II) by *Klebsiella* sp. Yangling I2. Initial pH = 5.0, biomass concentration = 1.0 g/L, and contact time = 20 h.

Several experiments were undertaken to study the effect of varying the initial ion concentration on the metal-removal process from the solution. The experimental results obtained using various ion concentrations between 0.5 and 20 mM for the biosorption of Cd(II) and Mn(II) onto *Klebsiella* sp. Yangling I2 are presented in Figs 8 and 9. Absorption capability increased continuously as the ion concentration increased, and saturation was achieved at 170.41 and 114.51 mg/g for Cd(II) and Mn(II), respectively. The maximum Cd(II) and Mn(II) removal percentages from the aqueous solution were 76.98% and 18.67% after 20 and 26 h at initial concentrations of 0.5 mM Cd(II) and 3 mM Mn(II). This curve clearly shows that as the initial ion concentration increased, an abrupt increase in the absorption capacity occurred and lower amounts of Cd(II) and Mn(II) were removed by the biosorbents. This behavior might be explained by the fact that the bacteria are stressed by the high concentration of heavy metals. Although the bacteria were stressed by the high heavy metal concentrations, more ions were bound to the surface, which can be explained as chemical reactions.

#### The effect of agitation speed on biosorption

**Fig 10:**
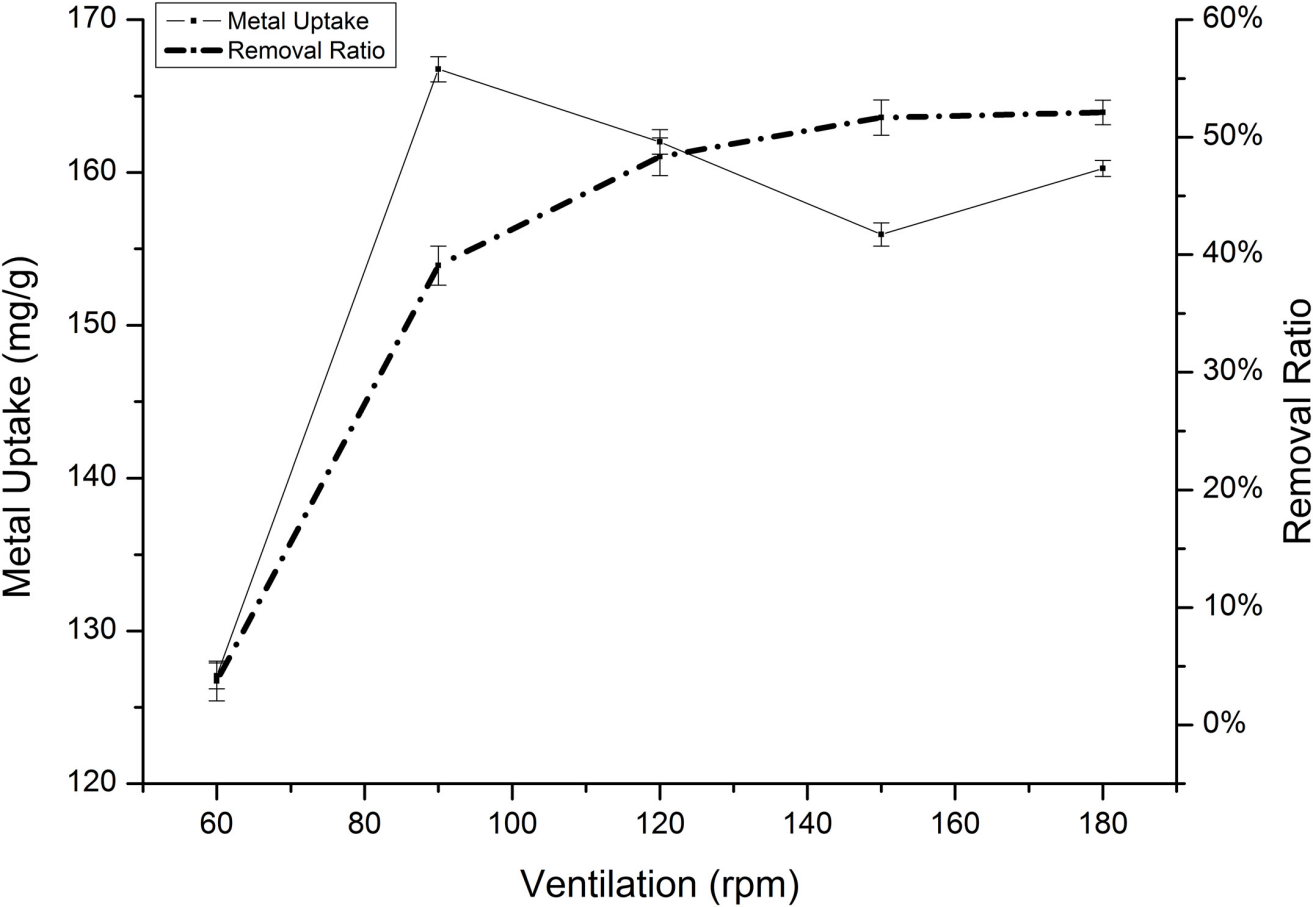
Effect of agitation speed on the absorption of Cd(II) by *Klebsiella* sp. Yangling I2. Initial ion concentration = 2 mM, initial pH = 5.5, and contact time = 26 h.

**Fig 11:**
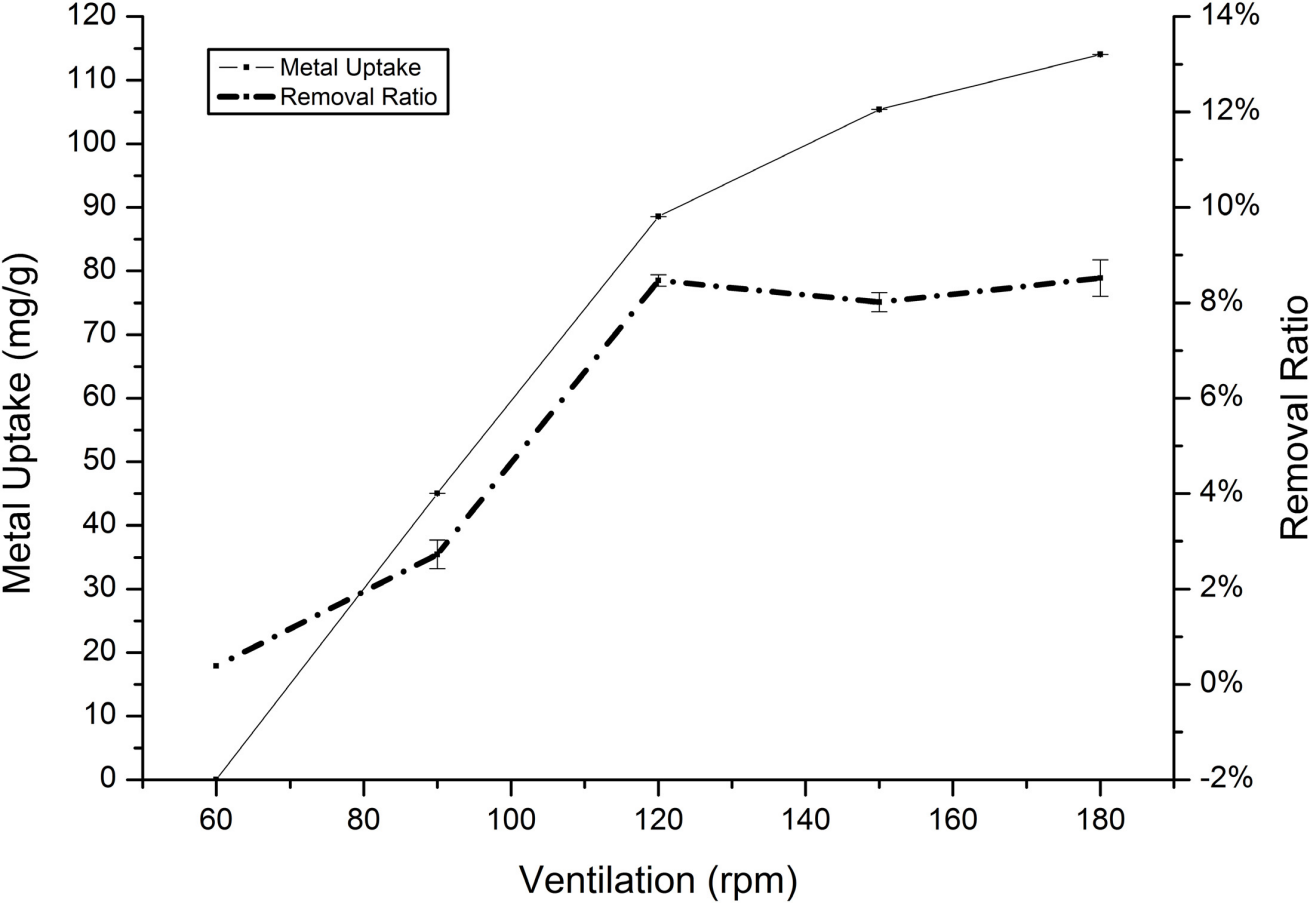
Effect of agitation speed on the absorption of Mn(II) by *Klebsiella* sp. Yangling I2. Initial ion concentration = 10 mM, initial pH = 5.0, and contact time = 20 h.

Agitation speed is the speed at which the solution is shaken during incubating. To determine the optimal agitation speed, cadmium and manganese removal experiments were shaken at rates between 60 rpm and 180 rpm. Fig 10 shows that the highest removal of cadmium at equilibrium is approximately 166.76 mg/g and was obtained with an agitation speed of 80 rpm, whereas the highest manganese removal was achieved at 120 rpm (Fig 11). At slow weak agitation speeds, we observed a reduction in the manganese sorption capacity by 114 mg/g to almost 0 mg/g. Additionally, when a high agitation speed was used, we noticed a substantial reduction, similar to the results reported by other authors [4].

#### The effect of biosorbent density on biosorption

**Fig 12:**
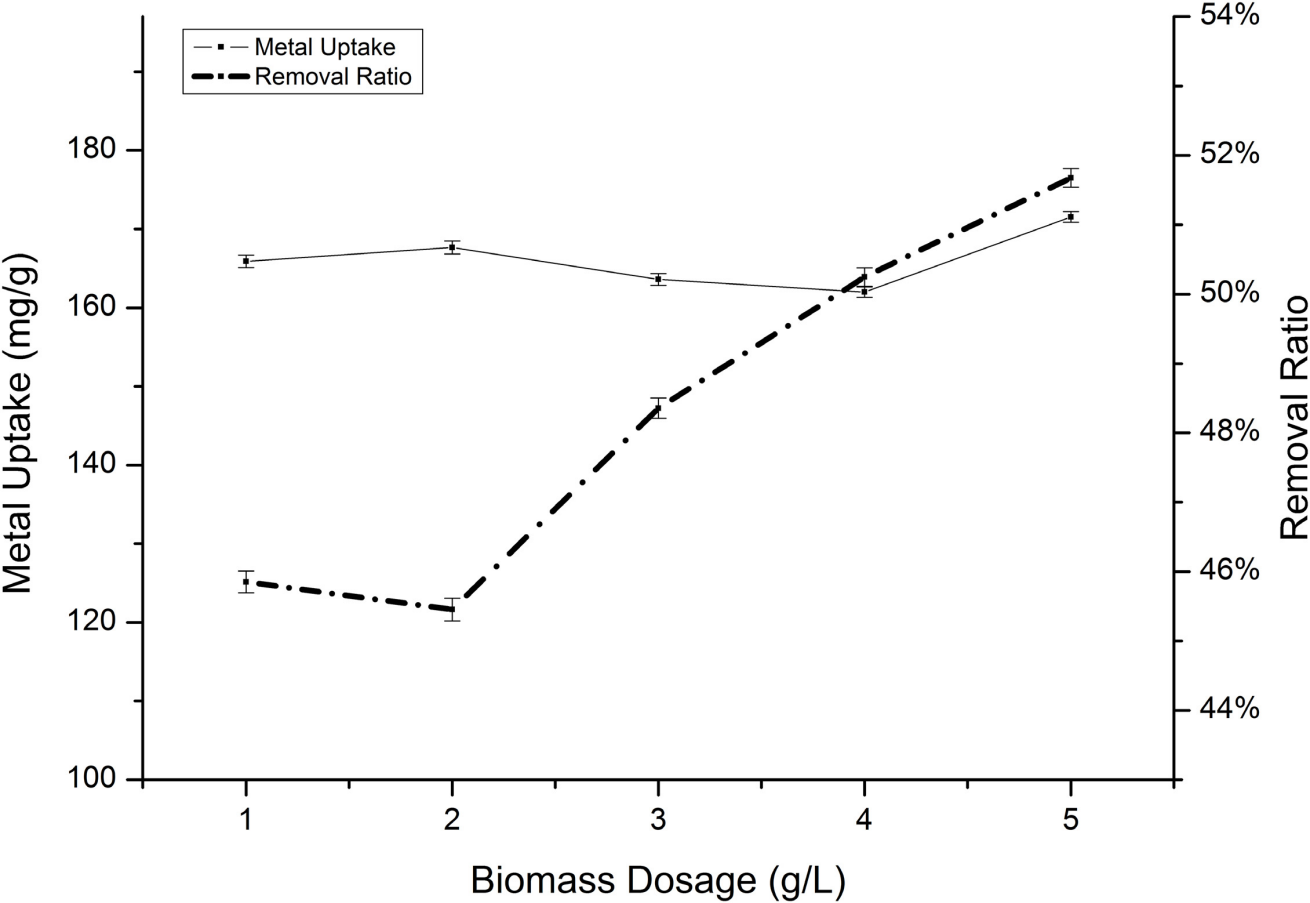
Effect of biosorbent density on the absorption of Cd(II) by *Klebsiella* sp. Yangling I2. Initial ion concentration = 2 mM, initial pH = 5.5, and contact time = 26 h.

**Fig 13:**
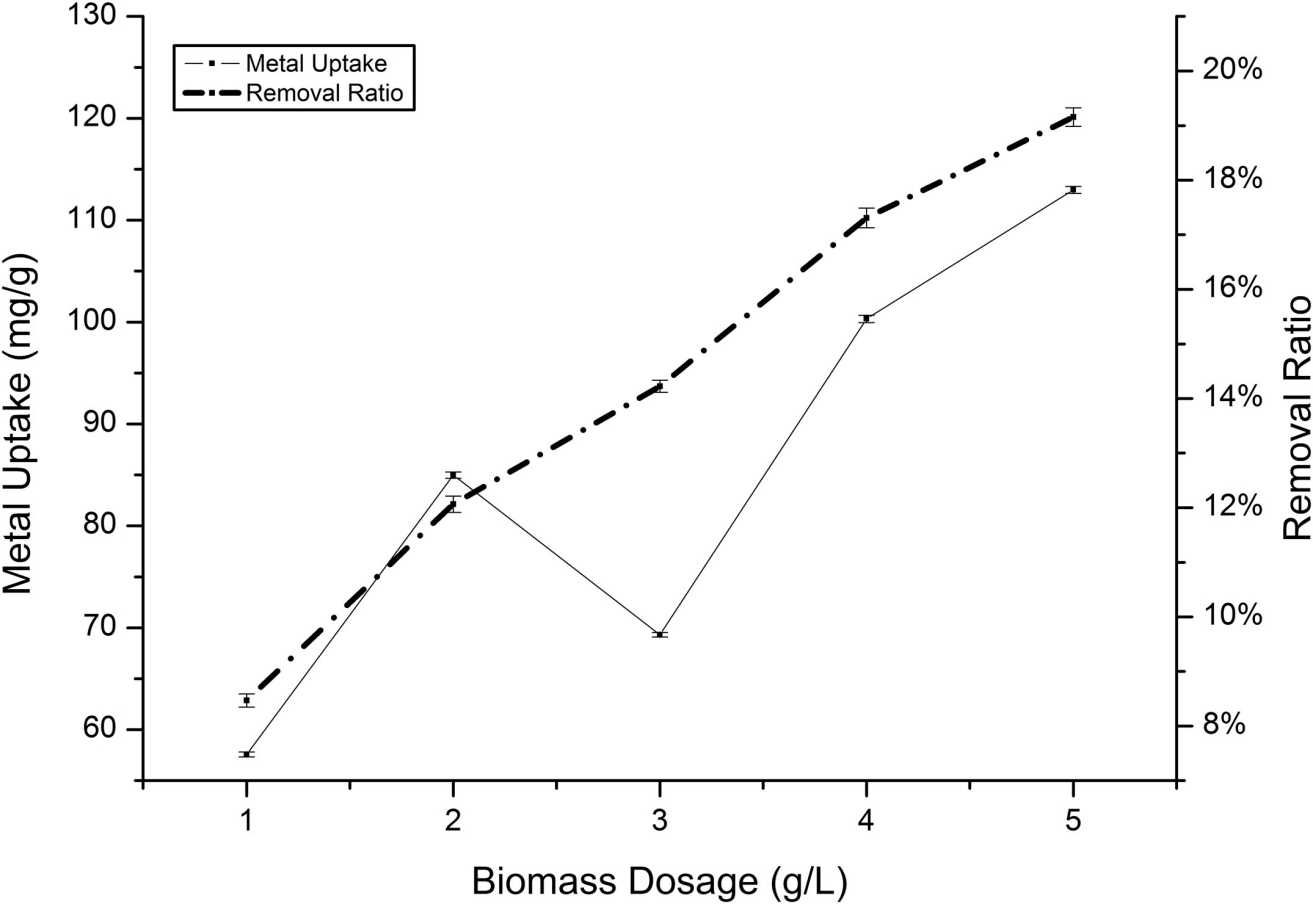
Effect of biosorbent density on the absorption of Mn(II) by *Klebsiella* sp. Yangling I2. Initial ion concentration = 10 mM, initial pH = 5.0, and contact time = 20 h.

The influence of biomass concentration on Cd(II) and Mn(II) absorption by *Klebsiella* sp. Yangling I2 was estimated using biosorbent doses ranging from 1.0 to 5.0 g/L. The ion removal efficiency was observed to be substantially enhanced as the biosorbent dosage increased, which could be attributed to increases in the absorption surface area and the availability of free absorption sites in the biomass (Fig 12). A substantial increase in the removal ratio was observed to result in a slight increase in the absorption capacity (Fig 13). This can be explained by the fact that as the mass increases, the available surface area for the sorption of cadmium and manganese also increases.

### The effect of contact time on biosorption

**Fig 14:**
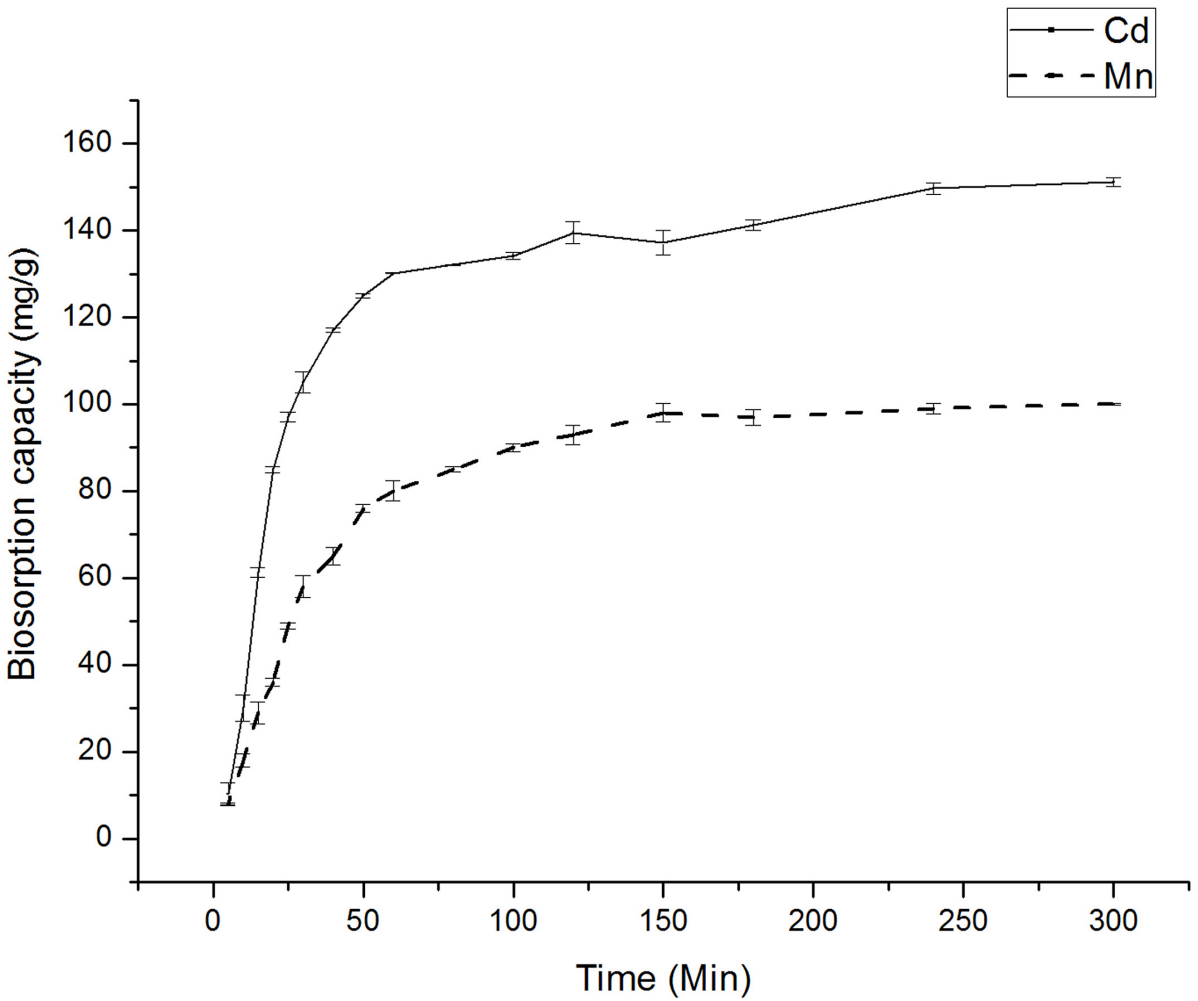
Effect of contact time on the uptake of Cd(II) and Mn(II) by *Klebsiella* sp. Yangling I2.

The effect of equilibrium time on the absorption of cadmium and manganese by *Klebsiella* sp. Yangling I2 is shown in Fig 14. For both Cd(II) and Mn(II), the absorption process can be divided into 2 stages: a rapid absorption process and a long, slow uptake process. In the rapid initial absorption stage, approximately 70% of the total ion uptake can be achieved because this process is spontaneous and does not consume energy. Rather, it is a type of non-metabolic-dependent ion sequestering on the surface of the biomass, which occurs through the mechanisms of physical absorption, ion exchange, and chemical complexion with the functional groups on the surface of the organism [40]. Subsequently, nearly 30% of the Cd and Mn ion absorption occurs in the second, slower period because the ions diffuse into the intracellular area of the organism [40]. Forces between the solute molecules of the solid and bulk phases can also prevent Cd and Mn ions from occupying the remaining vacant surface sites, contributing to the lower absorption rate in the latter stage [41,42]. Approximately 90% of Mn ion uptake was accomplished at 120 min, while Cd ion uptake required 180 min. These results are in agreement with the two-phase biosorption of heavy metals using different biomaterials [43,44].

### Biosorption equilibrium

The Langmuir and Freundlich sorption models are commonly used to fit experimental results when solute uptake occurs by monolayer sorption. These models were tested in the present work and permitted us to determine the maximal removal capacity.

Linear regression is a statistical method of modeling the relationship between two variables by fitting a linear equation to the observed data. One variable is considered to be an explanatory variable, and the other is considered to be a dependent variable. The linear regression method can be used for forecasting if it is assumed that the correlation between the variables will continue in the future [45]. However, models based on statistical regression are problematic if the data set is too small. if there is difficulty verifying that the error is normally distributed, or if there is vagueness in the relationship between the independent and dependent variables [46]. We reevaluated the Langmuir and Freundlich models based on fuzzy linear regression.

We focused on models for which the data are crisp and the relationship between the variables is fuzzy, and we propose a new fuzzy linear regression method for the equilibrium model. This proposed fuzzy linear regression is based on Tanaka’s approach using T_W_-based fuzzy arithmetic operations where the output data are fuzzy numbers [47]. Eq (4) is presented below.

$$s.t.\left\{\begin{array}{l} \text{min}J=c_{0}+c_{1}+\cdots c_{n}\\ a_{0}+\sum_{j}a_{j}x_{ij}-(1-H)(c_{0}+\sum_{j}c_{j}\left|x_{ij}\right|)\geq \tilde{y_{i}}\\ a_{0}+\sum_{j}a_{j}x_{ij}+(1-H)(c_{0}+\sum_{j}c_{j}\left|x_{ij}\right|)\leq \tilde{y_{i}}\\ c_{j}\geq 0,j=0,1,\cdots n \end{array}\right\}$$ (4)

where H is the h-certain factor.

**Fig 15:**
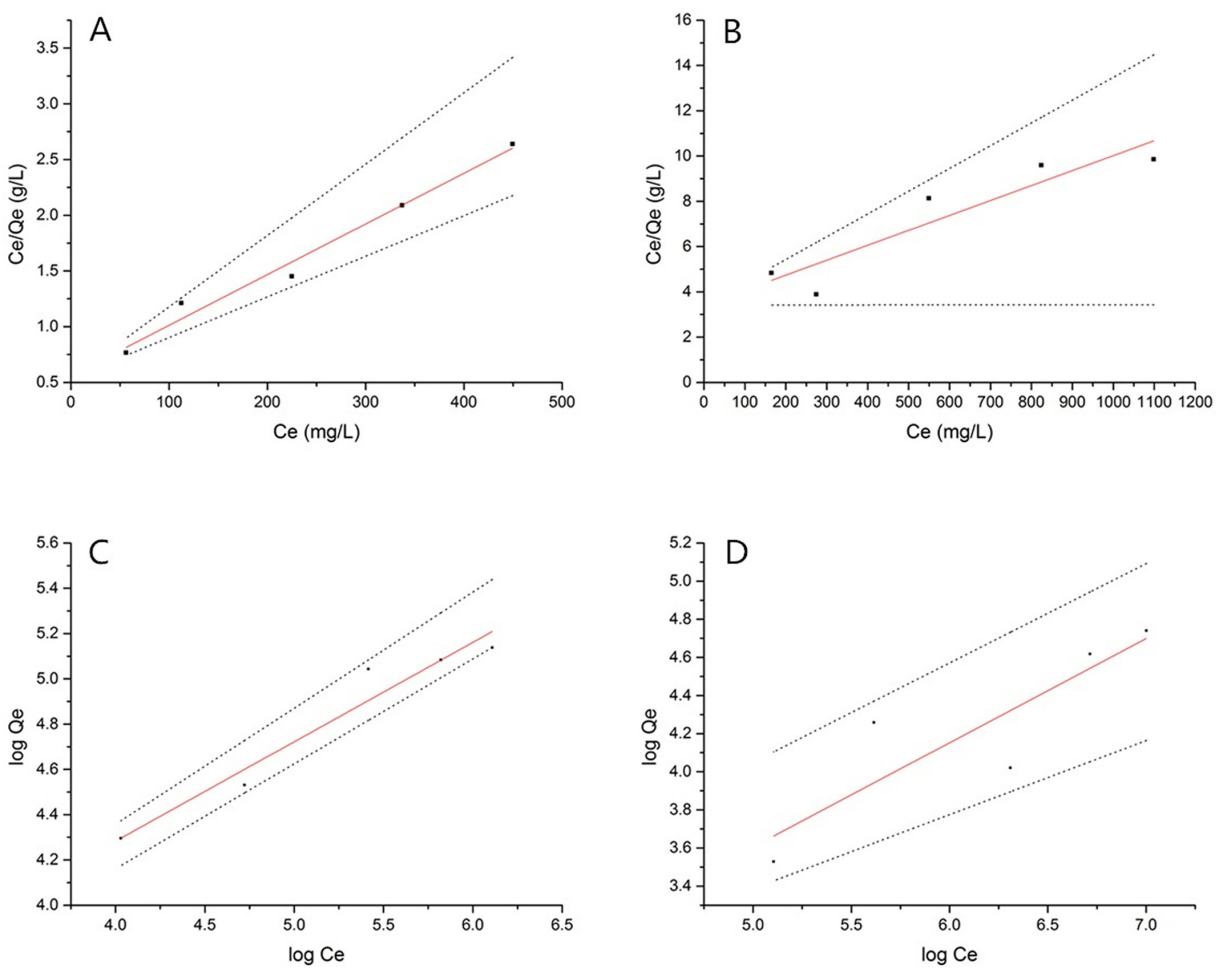
(A) Langmuir isotherm based on fuzzy linear regression for the absorption of cadmium. (B) Langmuir isotherm based on fuzzy linear regression for the absorption of manganese. (C) Freundlich isotherm based on fuzzy linear regression for the absorption of cadmium. (D) Freundlich isotherm based on fuzzy linear regression for the absorption of manganese.

The isotherm experimental results are shown in Fig 15A–15D. In all cases, favorable isotherms are observed.

#### Langmuir isotherm based on fuzzy linear regression

This monolayer absorption gives the maximum absorption capacity q_0_ in the linearized Langmuir expression, and the model can be described in terms of the following equation:

Langmuir isotherm [29,48,49]

CeQe=1q0b+Ceq0 (5)

where q_0_ and the constant b (absorption energy) are obtained from the slope and intercept of the plot of C_e_/Q_e_ against equilibrium concentration. C_e_ Fig 15A and 15B and the absorption energy are shown in Table 1. The statistical regression coefficients of cadmium and manganese were 0.9814 and 0.8574, respectively.

**Table 1:** Isotherm parameters obtained from the Langmuir model.

Metal	Isotherm model	Parameters	Values
Cadmium	Langmuir	q_0_	[156.21, 274.35]
		b	[0.0068, 0.0119]
		R_L_	[0.599, 0.724]
		R^2^	0.9814
Manganese	Langmuir	q_0_	[99.46, 718.58]
		b	[0.00037, 0.0029]
		R_L_	[0.674, 0.941]
		R^2^	0.8574

In this case, R_L_is a dimensionless parameter related to the effectiveness of metal absorption given by Eq (6) below:

$$R_{L}=\frac{1}{1+bC_{0}}$$ (6)

R_L_ values in the range of 0–1 indicate that the absorption process is effective [50]. The values of R_L_ for the absorption of cadmium (C_0_ = 56 mg/L) and manganese (C_0_ = 164 mg/L) were found to be [0.599, 0.724] and [0.674, 0.941]. This indicates the efficacy of the interaction between the microbial cell surface and divalent cadmium and manganese ion under the optimized experimental conditions.

The Langmuir model was able to fit the isotherm data with a high correlation coefficient. Comparing the q_0_ and R_L_ values (Table 1), Cd(II) biosorption is found to be superior to Mn(II) biosorption.

#### Freundlich isotherm based on fuzzy linear regression

The model is represented as Eq (7): [29,30]

logqe=logKf+1nlogCe (7)

where K_f_ is a Freundlich constant relating to the binding capacity and 1/n is an empirical parameter relating to the biosorption intensity, which varies according to the heterogeneity of the biosorbents. An efficient absorption process yields a Freundlich constant n in the range of 1 to 10. A high value of n implies a stronger interaction between the adsorbent cell surface and divalent metals. The logarithmic plot of q_e_ against C_e_ (Fig 15C and 15D) gives the constants K_f_ and n for the absorption (shown in Table 2).

**Table 2:** Isotherm parameters obtained from the Freundlich model.

Metal	Isotherm model	Parameters	Values
Cadmium	Freundlich	K_f_	10.04
		n	[1.95, 2.16]
		R^2^	0.9498
Manganese	Freundlich	K_f_	4.25
		n	[1.92, 2.58]
		R^2^	0.7721

### Comparison with other strains

The absorption capacity of the developed method was compared against other bacterial and fungal strains. The comparison (Table 3) shows that *Klebsiella* sp. has significant absorption capacity when compared with other strains. Hence, the novel *Klebsiella* sp. is effective at binding cadmium and manganese on its surface, and its application is facilitated by its high resistance to heavy metals.

**Table 3:** Absorption capacity comparison with various bacterial and fungal strains.

Metal	Strain	Absorption capacity (mg/g)	Reference
Cadmium	*Bacillus circulans*	26.5	[51]
	*Enterobacter* sp. J1	46.2	[52]
	*Pseudomonas aeruginosa* PU21	42.4	[53]
	*Pseudomonas putida*	8.0	[54]
	*Streptomyces pimprina* ^*a*^	30.4	[55]
	*Klebsiella* sp. Yangling I2	170.4	Present study
Manganese	*Klebsiella* sp. Yangling I2	114.1	Present study

## Conclusion

Because microorganisms lose viability in the presence of high concentrations of toxic heavy metal ions, the isolation of metal-reducing bacteria from contaminated environments is significant. The present study indicated that the strain *Klebsiella* sp. Yangling I2 has the ability to tolerate moderately high concentrations of heavy metal ions and exhibits relatively high, previously unreported biomass uptake (170.4 and 114.1 mg/g for Cd(II) and Mn(II), respectively). *Klebsiella* sp. is a newly discovered bacterium that was examined physically and biochemically. Based on 16s rRNA and whole genome sequencing, we believe that it is a new species. The surface characterization of the adsorbent also showed binding of the cadmium ion onto the surface of the adsorbent. Several factors, such as pH, temperature, initial metal concentration, agitation speed, and biomass density, were found to have a profound effect on Cd(II) and Mn(II) absorption. According to our experiment, we believe that the process of biosorption can be explained as a chemical reaction between ions and chemical groups on the surface of biomass. The Langmuir isotherm based on fuzzy linear regression was close to the curve and yielded binding constants of 0.98 and 0.86 for Cd and Mn, respectively.

The main finding of this study is that *Klebsiella* sp. Yangling I2 can adsorb approximately 10 times more cadmium than previously reported adsorbents, and the biosorption equilibrium was determined based on fuzzy linear regression. Overall, this novel bacterium is able to remove cadmium at concentrations up to 400 mg/L, thus making the metal-removal process for environmental remediation both economical and green.
