# Correction: Biosorption of Cadmium and Manganese Using Free Cells of *Klebsiella* sp. Isolated from Waste Water

**DOI:** 10.1371/journal.pone.0198309

**Published:** 2018-05-24

**Authors:** Yunnan Hou, Keke Cheng, Zehua Li, Xiaohui Ma, Yahong Wei, Lei Zhang, Yao Wang

The strain identified in this study was classified incorrectly and appears incorrectly throughout the article. The original classification of this strain was performed using classical Sanger sequencing with the commonly used universal primers 27F and 1492R in June 2012. Based on the sequencing results, Yangling I2 was identified as *Klebsiella* sp.

However, the authors noted there was a mistake in the classification of this strain after the whole genome sequencing of the strain Yangling I2 in January 2016. The authors further analyzed the 16S sequence using SMRT sequencing data, and discovered that the Yangling I2 strain should be classified as *Raoultella ornithinolytica*. All instances of *Klebsiella* sp. should be corrected to *Raoultella* sp. The authors apologize for the error.

The authors submitted a revision of the sequence to NCBI under accession number JX196956, which can be viewed here: http://www.ncbi.nlm.nih.gov/nuccore/JX196956.

The complete genome sequence of Yangling I2 has been deposited in GenBank under the accession no. CP013338, CP013339 and CP013340 for the circular chromosome and two plasmids of this strain, respectively. The sequence deposited in GenBank can be viewed here: https://www.ncbi.nlm.nih.gov/nuccore/CP013338.

For further questions, please contact the corresponding author at yahongwei@nwafu.edu.cn.
